# 3,5-Bis(4-meth­oxy­phen­yl)-1-phenyl-4,5-dihydro-1*H*-pyrazole

**DOI:** 10.1107/S1600536811000687

**Published:** 2011-01-12

**Authors:** Zeliha Baktır, Mehmet Akkurt, S. Samshuddin, B. Narayana, H. S. Yathirajan

**Affiliations:** a Department of Physics, Faculty of Sciences, Erciyes University, 38039 Kayseri, Turkey; b Department of Studies in Chemistry, Mangalore University, Mangalagangotri 574 199, India; c Department of Studies in Chemistry, University of Mysore, Manasangotri, Mysore 570 006, India

## Abstract

In the title compound, C_23_H_22_N_2_O_2_, the central pyrazole ring is nearly planar (r.m.s. deviation = 0.046 Å) and it makes a dihedral angle of 18.5 (2)° with the phenyl ring. The dihedral angles between the phenyl and the two meth­oxy-substituted phenyl rings are 26.2 (2) and 80.6 (2)°. The crystal structure is stabilized by C—H⋯π stacking inter­actions and weak π–π inter­actions [centriod–centroid distance = 3.891 (2) Å].

## Related literature

For the biological activity of pyrazoline derivatives, see: Amir *et al.* (2008 ▶); Hes *et al.* (1978 ▶); Manna *et al.* (2005 ▶); Regaila *et al.* (1979 ▶); Sarojini *et al.* (2010 ▶). For the use of pyrazoline derivatives in organic synthesis, see: Klimova *et al.* (1999 ▶); Bhaskarreddy *et al.* (1997 ▶). For the physical properties of pyrazoline derivatives, see: Wiley *et al.* (1958 ▶); Zhi-Yun *et al.* (1999 ▶). For related structures, see: Fun *et al.* (2010 ▶); Jasinski *et al.* (2010*a* ▶,*b* ▶); Samshuddin *et al.* (2010 ▶).
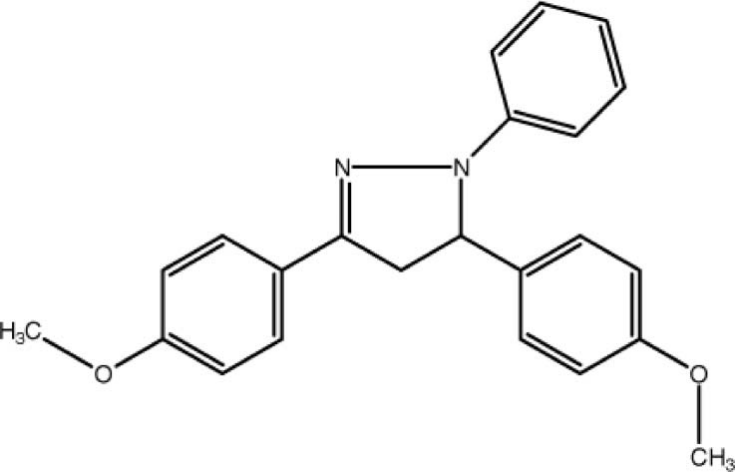


## Experimental

### 

#### Crystal data

C_23_H_22_N_2_O_2_
*M* *_r_* = 358.43
Monoclinic, 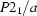

*a* = 9.4788 (5) Å
*b* = 10.1893 (6) Å
*c* = 19.9139 (10) Å
β = 92.296 (4)°
*V* = 1921.79 (18) Å^3^
*Z* = 4
Mo *K*α radiation
μ = 0.08 mm^−1^
*T* = 294 K
0.20 × 0.20 × 0.20 mm

#### Data collection

Rigaku R-AXIS RAPID-S diffractometer
Absorption correction: multi-scan (*SORTAV*; Blessing, 1995 ▶) *T*_min_ = 0.984, *T*_max_ = 0.984
33689 measured reflections
3191 independent reflections
1106 reflections with *I* > 2σ(*I*)
*R*_int_ = 0.224

#### Refinement

*R*[*F* ^2^ > 2σ(*F* ^2^)] = 0.062
*wR*(*F* ^2^) = 0.184
*S* = 0.90
3191 reflections
247 parameters
H-atom parameters constrained
Δρ_max_ = 0.14 e Å^−3^
Δρ_min_ = −0.20 e Å^−3^

### 

Data collection: *CrystalClear* (Rigaku/MSC, 2005 ▶); cell refinement: *CrystalClear*; data reduction: *CrystalClear*; program(s) used to solve structure: *SIR97* (Altomare *et al.*, 1999 ▶); program(s) used to refine structure: *SHELXL97* (Sheldrick, 2008 ▶); molecular graphics: *ORTEP-3 for Windows* (Farrugia, 1997 ▶); software used to prepare material for publication: *WinGX* (Farrugia, 1999 ▶).

## Supplementary Material

Crystal structure: contains datablocks global, I. DOI: 10.1107/S1600536811000687/su2245sup1.cif

Structure factors: contains datablocks I. DOI: 10.1107/S1600536811000687/su2245Isup2.hkl

Additional supplementary materials:  crystallographic information; 3D view; checkCIF report

## Figures and Tables

**Table 1:** Hydrogen-bond geometry (Å, °) *Cg*3 and *Cg*4 are the centroids of the phenyl (C11–C16) and benzene (C17–C22) rings, respectively.

*D*—H⋯*A*	*D*—H	H⋯*A*	*D*⋯*A*	*D*—H⋯*A*
C9—H9*A*⋯*Cg*3^i^	0.97	2.97	3.779 (5)	141
C14—H14⋯*Cg*4^ii^	0.93	2.68	3.588 (6)	167
C21—H21⋯*Cg*3^iii^	0.93	2.93	3.779 (4)	153
C23—H23*C*⋯*Cg*4^iii^	0.96	2.92	3.682 (5)	138

Symmetry codes: (i) 
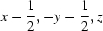
; (ii) 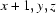; (iii) 
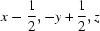
.

**Fig. 1.**
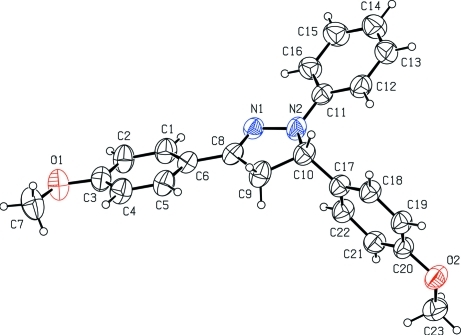
The molecular structure and numbering scheme for the title compound, with displacement ellipsoids drawn at the 30% probability level.

**Fig. 2.**
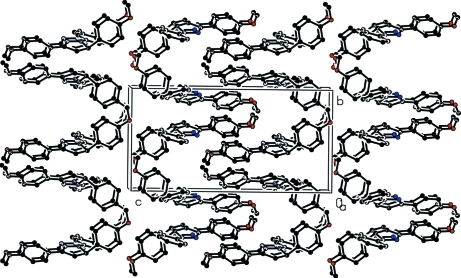
The crystal packing diagram of the title compound, viewed down the a axis (Hydrogen atoms have been omitted for clarity).

## supplementary crystallographic information

### Comment

Pyrazoline derivatives are well known for their versatile pharmacological activities such as antitumor, antibacterial, antifungal, antiviral, antiparasitic, anti-tubercular and insecticidal agents (Hes *et al.*, 1978; Manna *et al.*. 2005; Amir *et al.*, 2008). Some of these compounds have also anti-inflammatory, anti-diabetic, anaesthetic, analgesic and DPPH scavenging properties (Sarojini *et al.*, 2010; Regaila *et al.*., 1979). Because of their diverse properties, fairly assessable path of synthesis, wide range of therapeutic activities and variety of industrial application, the pyrazoline ring became a center of attraction for organic chemists. Several 1,3,5-triaryl-2-pyrazolines were also used as scintillation solutes (Wiley *et al.*, 1958). In addition, pyrazolines have played a crucial part in the development of theory in heterocyclic chemistry and also used extensively in organic synthesis (Klimova *et al.*., 1999 & Bhaskarreddy *et al.*, 1997). Many pyrazolines have excellent fluorescent property (Zhi-Yun *et al.*, 1999).

In continuation of our work on pyrazoline derivatives (Samshuddin *et al.*, 2010, Fun *et al.*, 2010, Jasinski *et al.*, 2010*a*,*b*) and in view of the importance of these derivatives, the title compound was synthesized and its crystal structure is reported on herein.

The title compound (Fig. 1), contains two methoxyphenyl groups (C1–C6 and C17–C22) and a phenyl ring (C11–C16) attached to the central pyrazole ring (N1/N2/C8–C10) which is nearly planar [r.m.s. deviation = 0.046 Å]. The dihedral angle between the two methoxy-substituted phenyl groups (C1–C6 and C17–C22) is 73.3 (2)°, and the dihedral angle between the pyrazole (N1/N2/C8–C10) and phenyl (C11–C16) rings is 18.5 (2)°. Also, the dihedral angles between the phenyl ring (C11–C16) and the two methoxy-substituted phenyl rings (C1–C6 and C17–C22) are 26.2 (2) and 80.6 (2)°, respectively.

The crystal structure is stabilized by C—H···π stacking interactions (Table 1) and weak π-π interactions [*Cg*1···*Cg*4(*x*, *y*, *z*) = 3.891 (2) Å, *Cg*1 and *Cg*4 are the centroids of the (N1/N2/C8–C10) pyrazole ring and the (C17–C22) benzene ring, respectively].

Fig. 2 shows the crystal packing diagram of of the title compound, viewed down the *a* axis. .

### Experimental

A mixture of (2*E*)-1,3-bis(4-methoxyphenyl)prop-2-en-1-one (2.68 g, 0.01 mol) and phenyl hydrazine (1.08 g, 0.01 mol) in 50 ml glacial acetic acid was refluxed for 6 h. The reaction mixture was cooled and poured into 50 ml of ice-cold water. The precipitate was collected by filtration and purified by recrystallization from ethanol. Yellow block-like crystals of the title compound were grown from toluene by slow evaporation (m. p.: 414 K, Yield: 76%).

### Refinement

The crystal diffracted weakly beyond 22° in θ, and only 35% of the data can be considerd to be observed [I>2σ(I)], hence the rather high R_int_ value. H-atoms were placed in geometrically idealized positions, with C—H distances in the range 0.93–0.98 Å, and refined as riding with *U*_iso_(H) = k× *U*_eq_(C), where k = 1.2 for methine, methylene and aromatic H-atoms and k = 1.5 for methyl H-atoms.

### Crystal data

**Table:**

C_23_H_22_N_2_O_2_	*F*(000) = 760
*M**_r_* = 358.43	*D*_x_ = 1.239 Mg m^−^^3^
Monoclinic, *P*2_1_/*a*	Mo *K*α radiation, λ = 0.71073 Å
Hall symbol: -P 2yab	Cell parameters from 2377 reflections
*a* = 9.4788 (5) Å	θ = 2.1–26.4°
*b* = 10.1893 (6) Å	µ = 0.08 mm^−^^1^
*c* = 19.9139 (10) Å	*T* = 294 K
β = 92.296 (4)°	Block, yellow
*V* = 1921.79 (18) Å^3^	0.20 × 0.20 × 0.20 mm
*Z* = 4	

### Data collection

**Table:**

Rigaku R-AXIS RAPID-S diffractometer	3191 independent reflections
Radiation source: Sealed Tube	1106 reflections with *I* > 2σ(*I*)
Graphite Monochromator	*R*_int_ = 0.224
Detector resolution: 10.0000 pixels mm^-1^	θ_max_ = 24.5°, θ_min_ = 2.1°
dtprofit.ref scans	*h* = −10→11
Absorption correction: multi-scan (*SORTAV*; Blessing, 1995)	*k* = −11→11
*T*_min_ = 0.984, *T*_max_ = 0.984	*l* = −23→23
33689 measured reflections	

### Refinement

**Table:**

Refinement on *F*^2^	Primary atom site location: structure-invariant direct methods
Least-squares matrix: full	Secondary atom site location: difference Fourier map
*R*[*F*^2^ > 2σ(*F*^2^)] = 0.062	Hydrogen site location: inferred from neighbouring sites
*wR*(*F*^2^) = 0.184	H-atom parameters constrained
*S* = 0.90	*w* = 1/[σ^2^(*F*_o_^2^) + (0.0524*P*)^2^] where *P* = (*F*_o_^2^ + 2*F*_c_^2^)/3
3191 reflections	(Δ/σ)_max_ < 0.001
247 parameters	Δρ_max_ = 0.14 e Å^−^^3^
0 restraints	Δρ_min_ = −0.20 e Å^−^^3^

### Special details

**Table:**

Geometry. Bond distances, angles *etc*. have been calculated using the rounded fractional coordinates. All su's are estimated from the variances of the (full) variance-covariance matrix. The cell e.s.d.'s are taken into account in the estimation of distances, angles and torsion angles
Refinement. Refinement on *F*^2^ for ALL reflections except those flagged by the user for potential systematic errors. Weighted *R*-factors *wR* and all goodnesses of fit *S* are based on *F*^2^, conventional *R*-factors *R* are based on *F*, with *F* set to zero for negative *F*^2^. The observed criterion of *F*^2^ > σ(*F*^2^) is used only for calculating -*R*-factor-obs *etc*. and is not relevant to the choice of reflections for refinement. *R*-factors based on *F*^2^ are statistically about twice as large as those based on *F*, and *R*-factors based on ALL data will be even larger.

### Fractional atomic coordinates and isotropic or equivalent isotropic displacement parameters (Å^2^)

**Table:**

	*x*	*y*	*z*	*U*_iso_*/*U*_eq_	
O1	0.0819 (4)	−0.1272 (3)	0.39060 (17)	0.1161 (16)	
O2	0.0782 (3)	0.1990 (3)	0.98107 (15)	0.0975 (12)	
N1	0.4111 (4)	−0.0467 (3)	0.67195 (17)	0.0765 (16)	
N2	0.4312 (4)	−0.0442 (3)	0.74168 (17)	0.0830 (16)	
C1	0.2937 (5)	−0.0382 (4)	0.5358 (2)	0.0921 (19)	
C2	0.2422 (5)	−0.0461 (4)	0.4715 (2)	0.099 (2)	
C3	0.1238 (5)	−0.1237 (5)	0.4573 (2)	0.091 (2)	
C4	0.0598 (5)	−0.1887 (5)	0.5072 (3)	0.097 (2)	
C5	0.1130 (5)	−0.1780 (4)	0.5723 (2)	0.091 (2)	
C6	0.2324 (5)	−0.1044 (4)	0.5883 (2)	0.0772 (17)	
C7	−0.0360 (6)	−0.2097 (5)	0.3715 (2)	0.129 (3)	
C8	0.2877 (5)	−0.0945 (4)	0.6573 (2)	0.0762 (17)	
C9	0.2094 (5)	−0.1353 (5)	0.7176 (2)	0.097 (2)	
C10	0.3152 (5)	−0.1076 (5)	0.7766 (2)	0.0836 (17)	
C11	0.5689 (5)	−0.0366 (4)	0.7691 (2)	0.0757 (17)	
C12	0.5991 (5)	−0.0710 (4)	0.8356 (2)	0.088 (2)	
C13	0.7370 (6)	−0.0628 (5)	0.8621 (3)	0.099 (2)	
C14	0.8423 (6)	−0.0196 (5)	0.8245 (3)	0.105 (3)	
C15	0.8142 (5)	0.0157 (5)	0.7582 (3)	0.108 (2)	
C16	0.6772 (5)	0.0088 (4)	0.7303 (2)	0.0897 (19)	
C17	0.2569 (4)	−0.0218 (5)	0.8307 (2)	0.0770 (17)	
C18	0.2257 (4)	−0.0745 (4)	0.8926 (2)	0.0807 (17)	
C19	0.1665 (4)	0.0022 (5)	0.9410 (2)	0.0811 (17)	
C20	0.1368 (4)	0.1318 (5)	0.9293 (2)	0.0757 (17)	
C21	0.1693 (4)	0.1872 (4)	0.8692 (2)	0.0832 (17)	
C22	0.2303 (4)	0.1093 (5)	0.8205 (2)	0.0850 (19)	
C23	0.0220 (5)	0.3240 (5)	0.9679 (2)	0.112 (2)	
H1	0.37300	0.01340	0.54510	0.1110*	
H2	0.28530	−0.00050	0.43740	0.1190*	
H4	−0.01950	−0.24010	0.49750	0.1160*	
H5	0.06760	−0.22140	0.60640	0.1100*	
H7A	−0.01370	−0.29920	0.38260	0.1920*	
H7B	−0.05570	−0.20230	0.32400	0.1920*	
H7C	−0.11730	−0.18260	0.39520	0.1920*	
H9A	0.18490	−0.22770	0.71550	0.1170*	
H9B	0.12390	−0.08410	0.72160	0.1170*	
H10	0.34820	−0.19080	0.79640	0.1000*	
H12	0.52710	−0.09970	0.86240	0.1060*	
H13	0.75660	−0.08750	0.90650	0.1180*	
H14	0.93370	−0.01360	0.84300	0.1260*	
H15	0.88730	0.04440	0.73210	0.1300*	
H16	0.65840	0.03430	0.68600	0.1080*	
H18	0.24480	−0.16240	0.90150	0.0970*	
H19	0.14650	−0.03460	0.98230	0.0970*	
H21	0.15110	0.27560	0.86100	0.1000*	
H22	0.25360	0.14740	0.77990	0.1020*	
H23A	0.09740	0.38440	0.96020	0.1680*	
H23B	−0.02940	0.35290	1.00570	0.1680*	
H23C	−0.04030	0.32020	0.92870	0.1680*	

### Atomic displacement parameters (Å^2^)

**Table:**

	*U*^11^	*U*^22^	*U*^33^	*U*^12^	*U*^13^	*U*^23^
O1	0.143 (3)	0.124 (3)	0.080 (2)	−0.027 (2)	−0.012 (2)	−0.005 (2)
O2	0.117 (2)	0.099 (2)	0.078 (2)	0.016 (2)	0.0221 (19)	−0.0041 (19)
N1	0.081 (3)	0.083 (3)	0.066 (2)	0.002 (2)	0.0104 (19)	−0.0017 (19)
N2	0.088 (3)	0.104 (3)	0.058 (2)	−0.005 (2)	0.016 (2)	−0.002 (2)
C1	0.102 (3)	0.102 (4)	0.073 (3)	−0.012 (3)	0.014 (3)	0.007 (3)
C2	0.123 (4)	0.106 (4)	0.068 (3)	−0.021 (3)	0.007 (3)	0.004 (3)
C3	0.107 (4)	0.094 (4)	0.071 (3)	0.000 (3)	−0.002 (3)	−0.010 (3)
C4	0.106 (4)	0.098 (4)	0.087 (4)	−0.016 (3)	0.011 (3)	−0.010 (3)
C5	0.109 (4)	0.091 (4)	0.075 (3)	−0.013 (3)	0.016 (3)	−0.005 (3)
C6	0.085 (3)	0.079 (3)	0.068 (3)	0.002 (3)	0.010 (3)	−0.010 (2)
C7	0.162 (5)	0.112 (4)	0.108 (4)	−0.024 (4)	−0.038 (4)	−0.001 (3)
C8	0.085 (3)	0.075 (3)	0.070 (3)	−0.002 (3)	0.020 (3)	−0.004 (2)
C9	0.110 (4)	0.106 (4)	0.077 (3)	−0.021 (3)	0.024 (3)	−0.008 (3)
C10	0.094 (3)	0.086 (3)	0.072 (3)	−0.001 (3)	0.019 (3)	0.005 (2)
C11	0.082 (3)	0.077 (3)	0.069 (3)	0.003 (2)	0.016 (3)	0.001 (2)
C12	0.097 (4)	0.096 (4)	0.073 (3)	0.007 (3)	0.015 (3)	0.001 (3)
C13	0.106 (4)	0.099 (4)	0.091 (4)	0.014 (3)	−0.007 (3)	0.001 (3)
C14	0.090 (4)	0.112 (4)	0.111 (5)	−0.002 (3)	−0.005 (3)	−0.005 (3)
C15	0.089 (4)	0.126 (4)	0.109 (4)	−0.002 (3)	0.008 (3)	0.014 (4)
C16	0.085 (3)	0.101 (4)	0.083 (3)	0.005 (3)	0.002 (3)	0.012 (3)
C17	0.084 (3)	0.080 (3)	0.068 (3)	−0.001 (3)	0.014 (2)	0.005 (2)
C18	0.096 (3)	0.076 (3)	0.071 (3)	0.006 (2)	0.014 (3)	0.007 (2)
C19	0.096 (3)	0.087 (3)	0.061 (3)	0.004 (3)	0.013 (2)	0.011 (2)
C20	0.084 (3)	0.084 (3)	0.060 (3)	0.004 (3)	0.013 (2)	0.001 (3)
C21	0.091 (3)	0.079 (3)	0.080 (3)	0.007 (3)	0.007 (3)	0.004 (3)
C22	0.095 (3)	0.091 (4)	0.070 (3)	−0.002 (3)	0.017 (3)	0.011 (3)
C23	0.123 (4)	0.108 (4)	0.105 (4)	0.036 (3)	0.006 (3)	−0.009 (3)

### Geometric parameters (Å, °)

**Table:**

O1—C3	1.372 (5)	C18—C19	1.378 (6)
O1—C7	1.437 (6)	C19—C20	1.368 (7)
O2—C20	1.373 (5)	C20—C21	1.370 (6)
O2—C23	1.401 (6)	C21—C22	1.396 (6)
N1—N2	1.394 (5)	C1—H1	0.9300
N1—C8	1.290 (6)	C2—H2	0.9300
N2—C10	1.474 (6)	C4—H4	0.9300
N2—C11	1.397 (6)	C5—H5	0.9300
C1—C2	1.354 (6)	C7—H7A	0.9600
C1—C6	1.391 (6)	C7—H7B	0.9600
C2—C3	1.393 (7)	C7—H7C	0.9600
C3—C4	1.357 (7)	C9—H9A	0.9700
C4—C5	1.376 (7)	C9—H9B	0.9700
C5—C6	1.384 (6)	C10—H10	0.9800
C6—C8	1.454 (6)	C12—H12	0.9300
C8—C9	1.496 (6)	C13—H13	0.9300
C9—C10	1.540 (6)	C14—H14	0.9300
C10—C17	1.509 (6)	C15—H15	0.9300
C11—C12	1.389 (6)	C16—H16	0.9300
C11—C16	1.389 (6)	C18—H18	0.9300
C12—C13	1.392 (7)	C19—H19	0.9300
C13—C14	1.346 (8)	C21—H21	0.9300
C14—C15	1.384 (8)	C22—H22	0.9300
C15—C16	1.394 (7)	C23—H23A	0.9600
C17—C18	1.387 (6)	C23—H23B	0.9600
C17—C22	1.373 (7)	C23—H23C	0.9600
			
O1···C15^i^	3.358 (7)	H7A···H4	2.3700
O1···H15^i^	2.6100	H7A···N1^vi^	2.9300
O2···H13^ii^	2.9100	H7A···H16^vi^	2.5400
O2···H19^iii^	2.8300	H7B···H15^i^	2.5500
N1···H1	2.6100	H7C···C4	2.7400
N1···H16	2.4900	H7C···H4	2.2800
N1···H7A^iv^	2.9300	H9A···C5	2.9500
N2···H22	2.7100	H9A···H5	2.4000
C12···C17	3.280 (6)	H9A···C11^ix^	2.8700
C15···O1^i^	3.358 (7)	H9A···C16^ix^	2.8800
C17···C12	3.280 (6)	H9B···C22	2.9400
C1···H4^v^	2.9900	H10···C12	2.7600
C4···H7A	2.7900	H10···H12	2.3000
C4···H7C	2.7400	H10···H18	2.3600
C5···H9A	2.9500	H10···C13^ix^	3.0400
C7···H15^i^	3.0500	H10···C14^ix^	3.0000
C7···H4	2.5300	H12···C10	2.5900
C7···H16^vi^	3.0600	H12···C17	2.7300
C9···H5	2.6900	H12···C18	2.9500
C10···H12	2.5900	H12···H10	2.3000
C11···H9A^v^	2.8700	H13···O2^ii^	2.9100
C12···H21^vii^	3.0900	H13···H18^v^	2.5500
C12···H10	2.7600	H14···C17^x^	3.0800
C13···H18^v^	2.9100	H14···C18^x^	2.9700
C13···H23A^vii^	3.0100	H14···C19^x^	2.8900
C13···H10^v^	3.0400	H14···C20^x^	2.9300
C13···H21^vii^	3.0400	H14···C21^x^	3.0600
C14···H10^v^	3.0000	H15···O1^i^	2.6100
C16···H9A^v^	2.8800	H15···C7^i^	3.0500
C17···H12	2.7300	H15···H7B^i^	2.5500
C17···H14^viii^	3.0800	H16···N1	2.4900
C18···H12	2.9500	H16···C7^iv^	3.0600
C18···H14^viii^	2.9700	H16···H7A^iv^	2.5400
C19···H14^viii^	2.8900	H16···H2^i^	2.5600
C20···H14^viii^	2.9300	H18···H10	2.3600
C21···H14^viii^	3.0600	H18···C13^ix^	2.9100
C21···H23A	2.8100	H18···H13^ix^	2.5500
C21···H23C	2.7200	H19···O2^iii^	2.8300
C21···H23C^vii^	2.9500	H21···C23	2.5500
C22···H9B	2.9400	H21···H23A	2.3400
C22···H23C^vii^	3.0800	H21···H23C	2.3500
C23···H21	2.5500	H21···C12^xi^	3.0900
H1···N1	2.6100	H21···C13^xi^	3.0400
H2···H16^i^	2.5600	H22···N2	2.7100
H4···C7	2.5300	H23A···C21	2.8100
H4···H7A	2.3700	H23A···H21	2.3400
H4···H7C	2.2800	H23A···C13^xi^	3.0100
H4···C1^ix^	2.9900	H23C···C21	2.7200
H5···C9	2.6900	H23C···H21	2.3500
H5···H9A	2.4000	H23C···C21^xi^	2.9500
H7A···C4	2.7900	H23C···C22^xi^	3.0800
			
C3—O1—C7	117.6 (4)	C3—C2—H2	121.00
C20—O2—C23	118.4 (3)	C3—C4—H4	120.00
N2—N1—C8	108.6 (3)	C5—C4—H4	120.00
N1—N2—C10	112.8 (3)	C4—C5—H5	119.00
N1—N2—C11	118.6 (3)	C6—C5—H5	119.00
C10—N2—C11	122.9 (3)	O1—C7—H7A	109.00
C2—C1—C6	122.4 (4)	O1—C7—H7B	109.00
C1—C2—C3	118.9 (4)	O1—C7—H7C	109.00
O1—C3—C2	114.3 (4)	H7A—C7—H7B	110.00
O1—C3—C4	125.2 (4)	H7A—C7—H7C	109.00
C2—C3—C4	120.6 (4)	H7B—C7—H7C	109.00
C3—C4—C5	119.6 (4)	C8—C9—H9A	111.00
C4—C5—C6	121.7 (4)	C8—C9—H9B	111.00
C1—C6—C5	116.9 (4)	C10—C9—H9A	111.00
C1—C6—C8	122.1 (4)	C10—C9—H9B	111.00
C5—C6—C8	121.0 (4)	H9A—C9—H9B	109.00
N1—C8—C6	122.0 (4)	N2—C10—H10	110.00
N1—C8—C9	113.5 (4)	C9—C10—H10	110.00
C6—C8—C9	124.6 (4)	C17—C10—H10	109.00
C8—C9—C10	103.5 (4)	C11—C12—H12	120.00
N2—C10—C9	101.3 (3)	C13—C12—H12	120.00
N2—C10—C17	112.6 (4)	C12—C13—H13	119.00
C9—C10—C17	113.9 (4)	C14—C13—H13	120.00
N2—C11—C12	120.8 (4)	C13—C14—H14	120.00
N2—C11—C16	120.1 (4)	C15—C14—H14	120.00
C12—C11—C16	119.1 (4)	C14—C15—H15	120.00
C11—C12—C13	120.1 (4)	C16—C15—H15	120.00
C12—C13—C14	121.0 (5)	C11—C16—H16	120.00
C13—C14—C15	119.8 (5)	C15—C16—H16	120.00
C14—C15—C16	120.5 (5)	C17—C18—H18	120.00
C11—C16—C15	119.5 (4)	C19—C18—H18	120.00
C10—C17—C18	120.5 (4)	C18—C19—H19	120.00
C10—C17—C22	121.9 (4)	C20—C19—H19	119.00
C18—C17—C22	117.6 (4)	C20—C21—H21	120.00
C17—C18—C19	120.6 (4)	C22—C21—H21	120.00
C18—C19—C20	121.0 (4)	C17—C22—H22	119.00
O2—C20—C19	116.1 (4)	C21—C22—H22	119.00
O2—C20—C21	124.2 (4)	O2—C23—H23A	109.00
C19—C20—C21	119.7 (4)	O2—C23—H23B	109.00
C20—C21—C22	119.1 (4)	O2—C23—H23C	109.00
C17—C22—C21	121.9 (4)	H23A—C23—H23B	109.00
C2—C1—H1	119.00	H23A—C23—H23C	109.00
C6—C1—H1	119.00	H23B—C23—H23C	110.00
C1—C2—H2	121.00		
			
C7—O1—C3—C2	177.4 (4)	C1—C6—C8—N1	−14.4 (7)
C7—O1—C3—C4	−2.3 (7)	C1—C6—C8—C9	164.6 (4)
C23—O2—C20—C19	−168.4 (4)	N1—C8—C9—C10	−2.3 (5)
C23—O2—C20—C21	13.3 (6)	C6—C8—C9—C10	178.7 (4)
N2—N1—C8—C9	−2.0 (5)	C8—C9—C10—C17	126.2 (4)
C8—N1—N2—C11	160.0 (3)	C8—C9—C10—N2	5.1 (4)
N2—N1—C8—C6	177.1 (4)	C9—C10—C17—C22	−70.6 (5)
C8—N1—N2—C10	5.8 (4)	N2—C10—C17—C18	−137.2 (4)
C10—N2—C11—C12	−10.0 (6)	N2—C10—C17—C22	44.1 (5)
N1—N2—C10—C9	−6.8 (5)	C9—C10—C17—C18	108.2 (5)
C11—N2—C10—C9	−159.6 (4)	N2—C11—C16—C15	−179.9 (4)
N1—N2—C10—C17	−128.8 (3)	C12—C11—C16—C15	1.7 (6)
C11—N2—C10—C17	78.3 (5)	N2—C11—C12—C13	−180.0 (4)
C10—N2—C11—C16	171.6 (4)	C16—C11—C12—C13	−1.6 (6)
N1—N2—C11—C12	−161.4 (4)	C11—C12—C13—C14	1.2 (7)
N1—N2—C11—C16	20.2 (5)	C12—C13—C14—C15	−0.9 (8)
C2—C1—C6—C8	−179.5 (4)	C13—C14—C15—C16	1.0 (8)
C2—C1—C6—C5	−1.2 (7)	C14—C15—C16—C11	−1.4 (7)
C6—C1—C2—C3	−0.2 (7)	C10—C17—C22—C21	176.5 (4)
C1—C2—C3—O1	−178.7 (4)	C18—C17—C22—C21	−2.3 (6)
C1—C2—C3—C4	1.0 (7)	C10—C17—C18—C19	−177.0 (4)
C2—C3—C4—C5	−0.3 (7)	C22—C17—C18—C19	1.8 (6)
O1—C3—C4—C5	179.5 (4)	C17—C18—C19—C20	0.1 (6)
C3—C4—C5—C6	−1.3 (7)	C18—C19—C20—C21	−1.5 (6)
C4—C5—C6—C1	2.0 (7)	C18—C19—C20—O2	−179.9 (3)
C4—C5—C6—C8	−179.7 (4)	O2—C20—C21—C22	179.2 (4)
C5—C6—C8—N1	167.5 (4)	C19—C20—C21—C22	1.0 (6)
C5—C6—C8—C9	−13.6 (7)	C20—C21—C22—C17	0.9 (6)

Symmetry codes: (i) −*x*+1, −*y*, −*z*+1; (ii) −*x*+1, −*y*, −*z*+2; (iii) −*x*, −*y*, −*z*+2; (iv) −*x*+1/2, *y*+1/2, −*z*+1; (v) *x*+1/2, −*y*−1/2, *z*; (vi) −*x*+1/2, *y*−1/2, −*z*+1; (vii) *x*+1/2, −*y*+1/2, *z*; (viii) *x*−1, *y*, *z*; (ix) *x*−1/2, −*y*−1/2, *z*; (x) *x*+1, *y*, *z*; (xi) *x*−1/2, −*y*+1/2, *z*.

### Hydrogen-bond geometry (Å, °)

**Table:**

Cg1, Cg3 and Cg4 are the centroids of the pyrazole (N1/N2/C8–C10), phenyl (C11–C16) and benzene (C17–C22) rings, respectively.

**Table:**

*D*—H···*A*	*D*—H	H···*A*	*D*···*A*	*D*—H···*A*
C9—H9A···Cg3^ix^	0.97	2.97	3.779 (5)	141
C14—H14···Cg4^x^	0.93	2.68	3.588 (6)	167
C21—H21···Cg3^xi^	0.93	2.93	3.779 (4)	153
C23—H23C···Cg4^xi^	0.96	2.92	3.682 (5)	138

Symmetry codes: (ix) *x*−1/2, −*y*−1/2, *z*; (x) *x*+1, *y*, *z*; (xi) *x*−1/2, −*y*+1/2, *z*.
